# Supplementary material for: Physiological and biochemical characterization of trypsin from Neocaridina denticulata sinensis and its roles in ontogenesis and immune response
Source: PLoS One. 2026 Feb 17;21(2):e0342746. doi: 10.1371/journal.pone.0342746 (PMC12912573; doi:10.1371/journal.pone.0342746)
Supplement: S1 File — (DOCX) [file pone.0342746.s001.docx]

**S1 File** Primers used in this study

| Primers | Sequences (5′ - 3′) | Sequence information |
| --- | --- | --- |
| NdTryp-ORF-F | ATGAAGACTCTAGTACTCTGCGTTC | Gene cloning |
| NdTryp-ORF-R | TTAGCCAGAGTTAGCAAGGACCCA | Gene cloning |
| NdTryp-qPCR-F | GTGCCTCCATCTACAATGAGAAC | Real-time PCR |
| NdTryp-qPCR-R | TCATCAACATCTAAGTTGTGCTCA | Real-time PCR |
| 18S rRNA-qPCR-F | GGGGAGGTAGTGACGAAAAAT | Real-time PCR |
| 18S rRNA-qPCR-R | TATATGCTATTGGAGCTGGAA | Real-time PCR |
| T7-NdTryp-F | TAATACGACTCACTATAGGGCGAGTTGTTGCTGGTGAGCACAAC | ISH-sense probe/dsRNA synthesis |
| NdTryp-R | AGTTAGCAAGGACCCAGTCAACGA | ISH-sense probe |
| NdTryp-F | CGAGTTGTTGCTGGTGAGCACAAC | ISH-antisense probe |
| T7-NdTryp-R | TAATACGACTCACTATAGGGAGTTAGCAAGGACCCAGTCAACGA | ISH-antisense probe/dsRNA synthesis |

**Note:** T7 promoter sequences are underlined.
