# Supplementary figures and images for: Physiological and biochemical characterization of trypsin from Neocaridina denticulata sinensis and its roles in ontogenesis and immune response
Source: PLoS One. 2026 Feb 17;21(2):e0342746. doi: 10.1371/journal.pone.0342746 (PMC12912573; doi:10.1371/journal.pone.0342746)

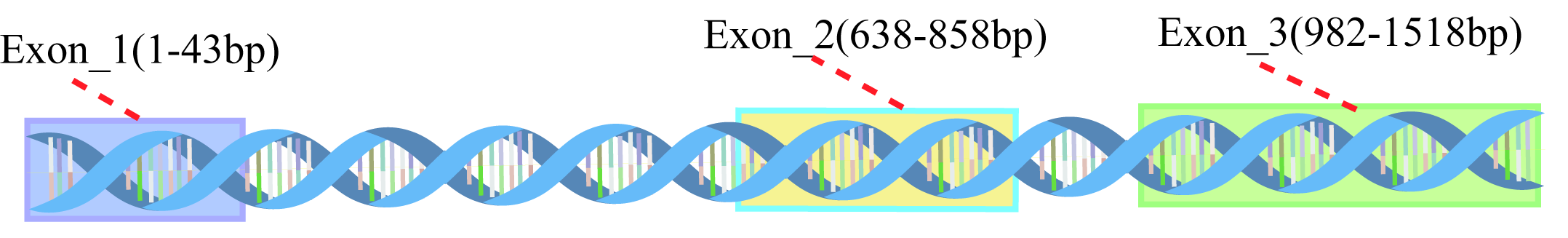


**S2 File** Genomic structure of *NdTryp* from *N. denticulata sinensis*.

Supplement: S2 File — (DOCX) [file pone.0342746.s002.docx]

**
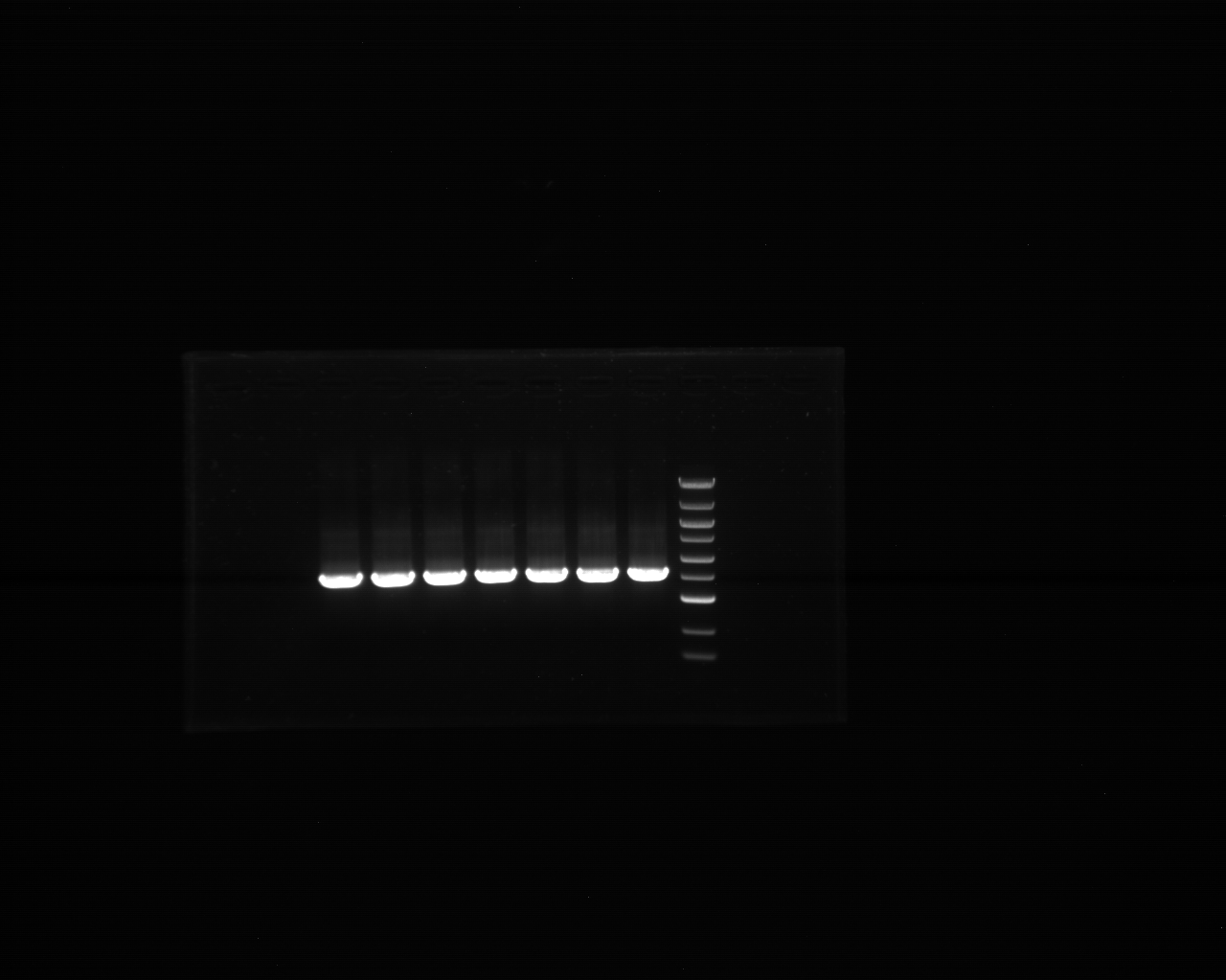
**

750 bp

**S4 File** Gel image of the ORF length of *NdTryp* amplified by NdTryp-ORF-F/R primers.

Supplement: S4 File — (DOCX) [file pone.0342746.s004.docx]

**
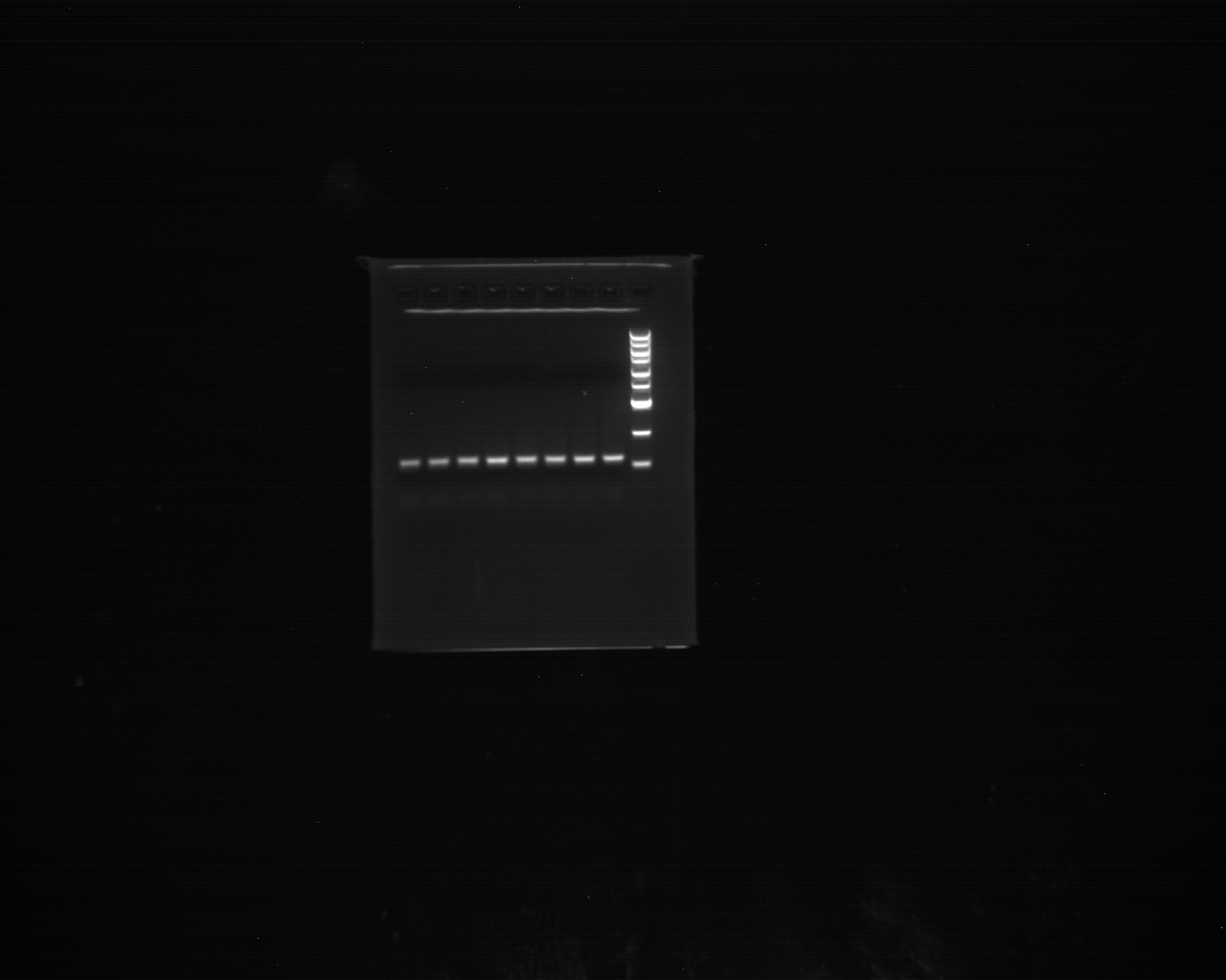
**

100 bp

**S6 File** Gel image of NdTryp-qPCR-F/R primers was used to verify the specificity of qPCR primers.

Supplement: S6 File — (DOCX) [file pone.0342746.s006.docx]

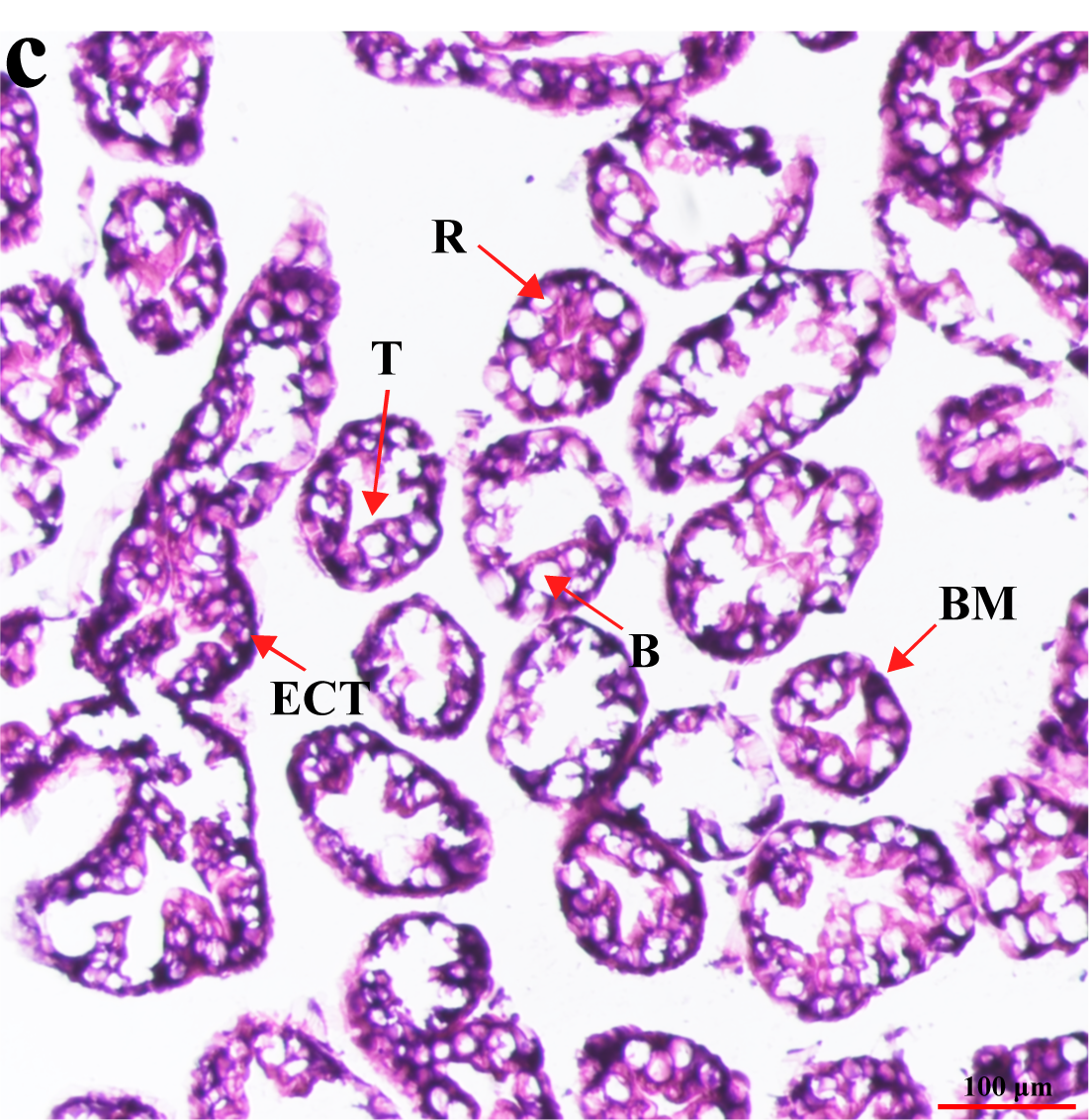

Supplement: S8 File — (TIF) [file pone.0342746.s008.tif]

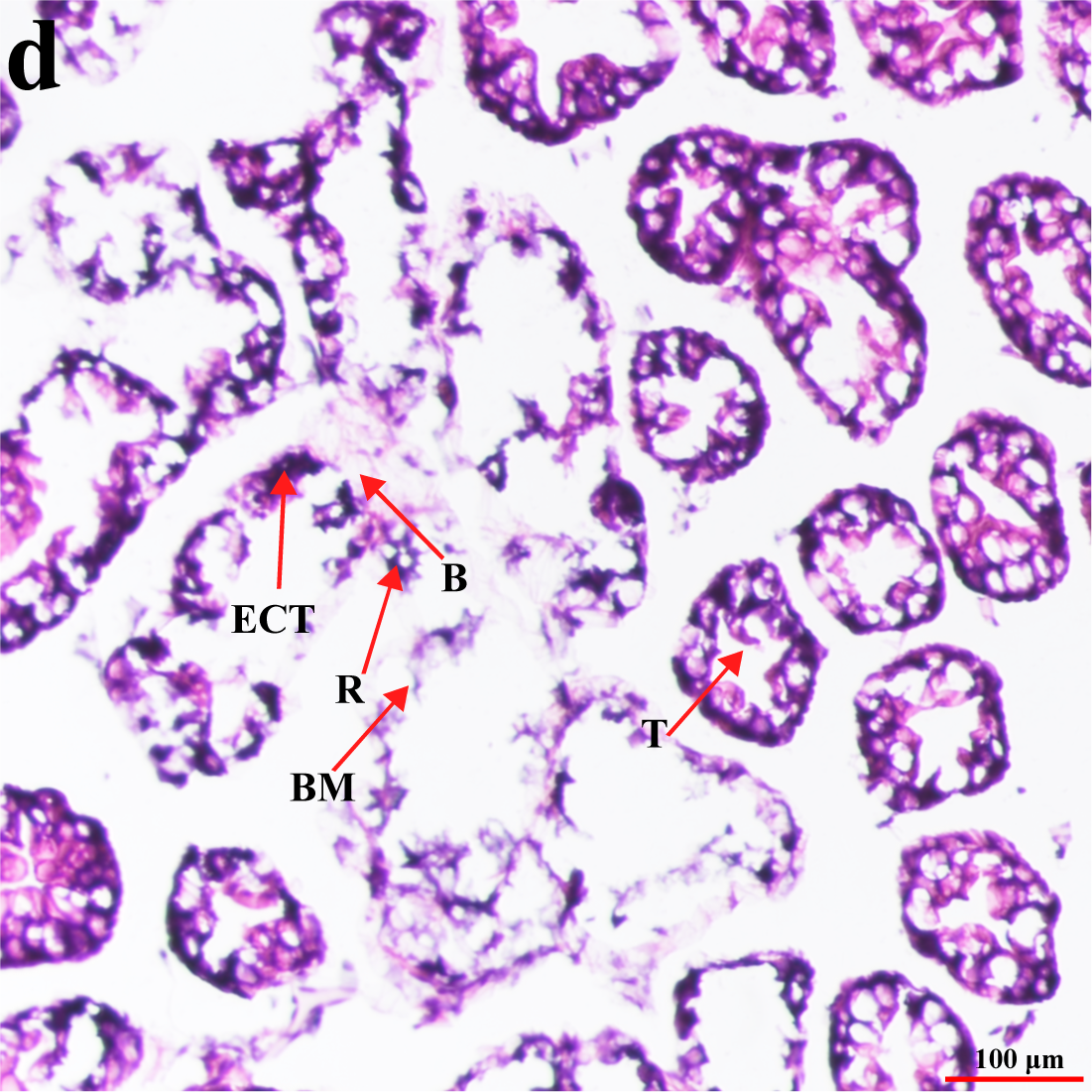

Supplement: S9 File — (TIF) [file pone.0342746.s009.tif]

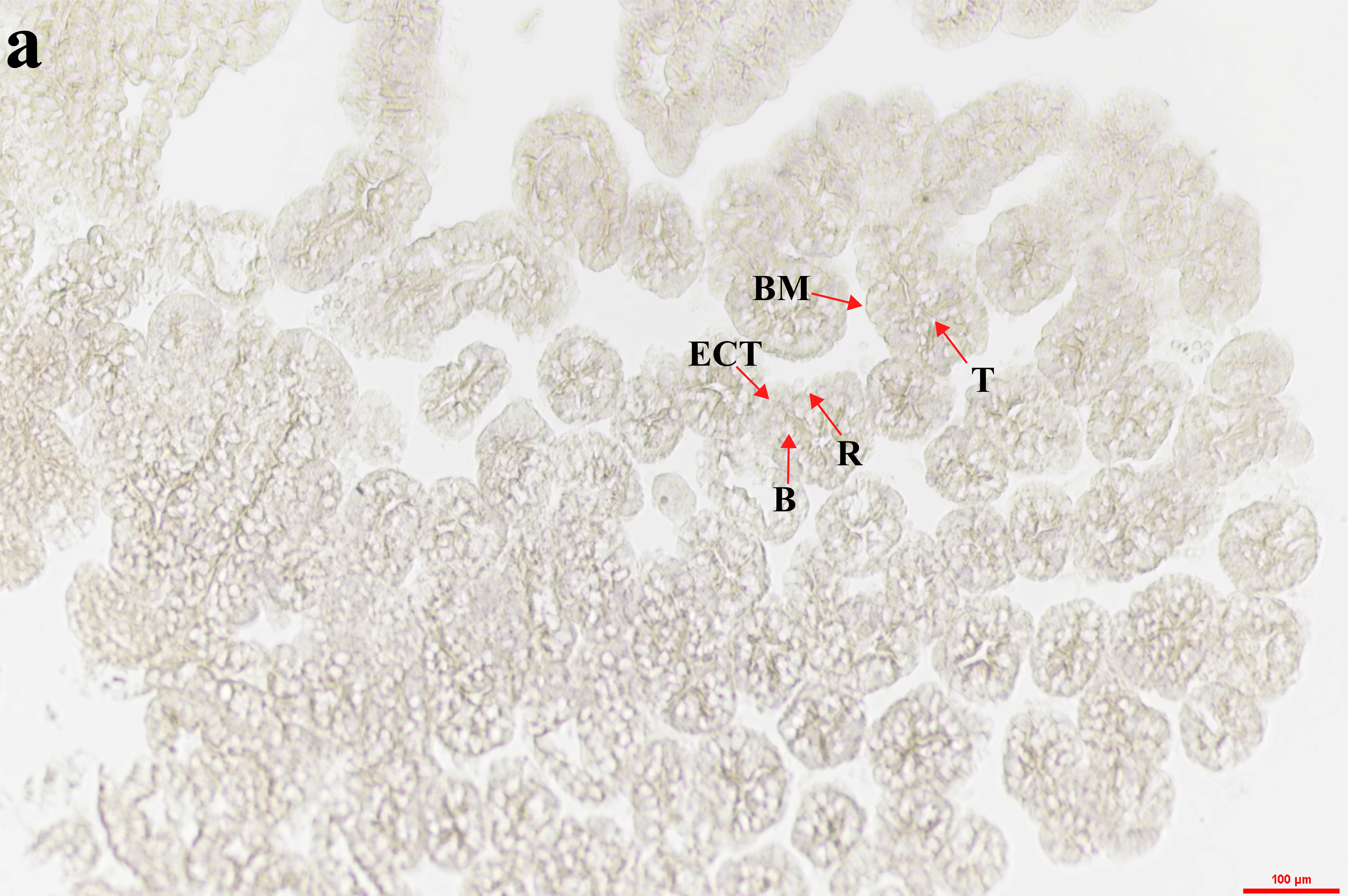

Supplement: S10 File — (TIF) [file pone.0342746.s010.tif]

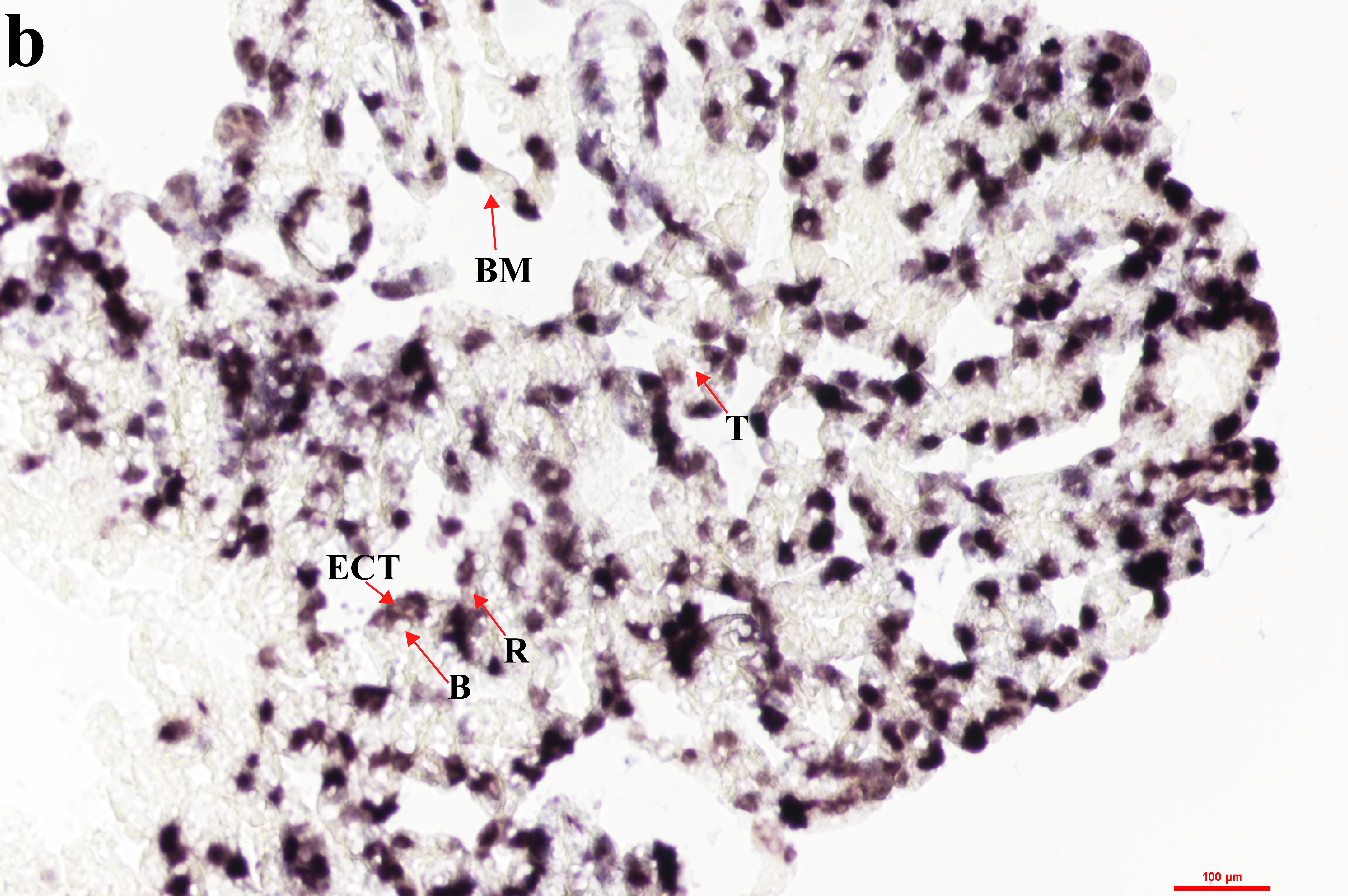

Supplement: S11 File — (TIF) [file pone.0342746.s011.tif]

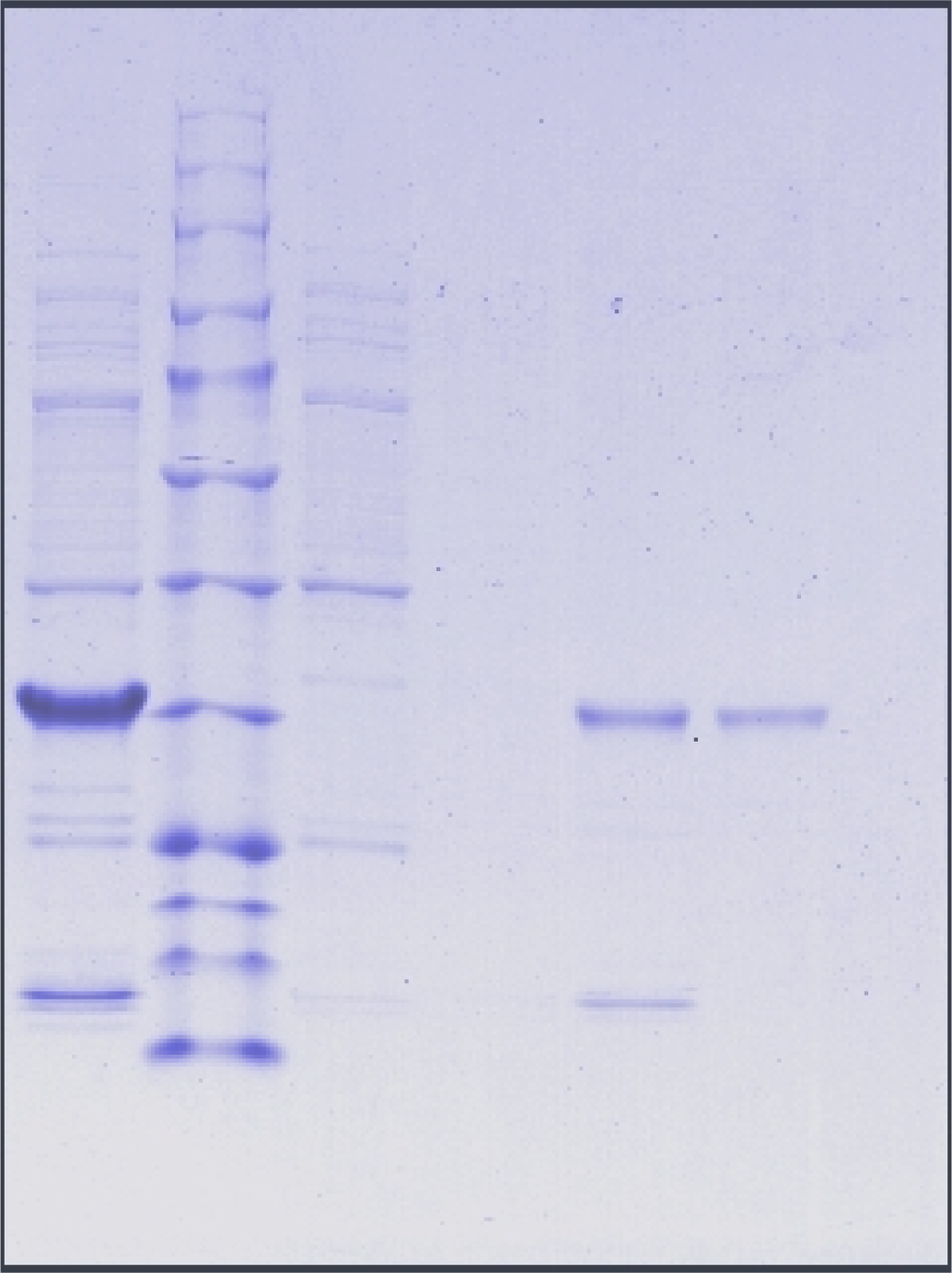

Supplement: S12 File — (TIF) [file pone.0342746.s012.tif]
