# Supplementary material for: Physiological and biochemical characterization of trypsin from Neocaridina denticulata sinensis and its roles in ontogenesis and immune response
Source: PLoS One. 2026 Feb 17;21(2):e0342746. doi: 10.1371/journal.pone.0342746 (PMC12912573; doi:10.1371/journal.pone.0342746)
Supplement: S3 File — (DOCX) [file pone.0342746.s003.docx]

**>Seq1 [organism=*Neocaridina denticulata sinensis*] trypsin (tryp) gene, complete cds**

ATTCATGTCTTCACCCTGGACCAGTGTCCGGCCATGAAGACTCTAGTACTCTGCGTTCTCCTTGCTGGGGCCTTTGCCGCCCCTTCCTCCAAGCCCCAGTTCCGTCGTGGACTGAACAAGATCGTCGGAGGATCTGAAGTTACTCCTGGTGACATCCCATACCAGCTTAGCTTCCAGGATATCTCCTTTGGTTCTCCATTCCACTTCTGTGGTGCCTCCATCTACAATGAGAACTGGGCCATCTGTGCTGGACACTGTGTCCAGGGTGAAGACATGAATAACCCTGACTACCTCCGAGTTGTTGCTGGTGAGCACAACTTAGATGTTGATGAGGGTAATGAACAGGCCATTGTTCTCTCAAGGATCATTCAACACGAGAATTACAATGGTTTCAGCATCAGCAACGACATCTCCCTTCTTCAGCTTTCTCAGCCTTTGACCTTCAACGACTTCGTTTCGGCCATTGCTCTTCCTGCAGCTGGTCACTCTGCCACTGGTGACTGTGTTGTCTCCGGATGGGGTACAACCAGTGAAGGAGGCTCTACCCCATCTTCCCTCATGGCTGTGACAGTACCCGTCGTCAGTGATGATGAATGTCGCGCTGCCTATGGTCAGACTGAGGTTGAAGACTCCATGATCTGCGCTGGTCTTCCTGAGGGAGGCAAGGACTCTTGCCAGGGAGATTCTGGTGGCCCAATGGTCTGCTCTGATACTGGATCCCCTTACCTGGCTGGTATTGTATCCTGGGGATATGGTTGTGCCCGCCCCAACTACCCAGGTGTCTACTGCGAGGTTTCTTACTTCGTTGACTGGGTCCTTGCTAACTCTGGCTAACTTTGATATCCGAGCCTGACTTTACCCACAGAAAAATAAATATTACTTAACAACAAAAAAAAAAAAAAAAAA
