# Supplementary material for: Physiological and biochemical characterization of trypsin from Neocaridina denticulata sinensis and its roles in ontogenesis and immune response
Source: PLoS One. 2026 Feb 17;21(2):e0342746. doi: 10.1371/journal.pone.0342746 (PMC12912573; doi:10.1371/journal.pone.0342746)
Supplement: S5 File — (DOCX) [file pone.0342746.s005.docx]

**
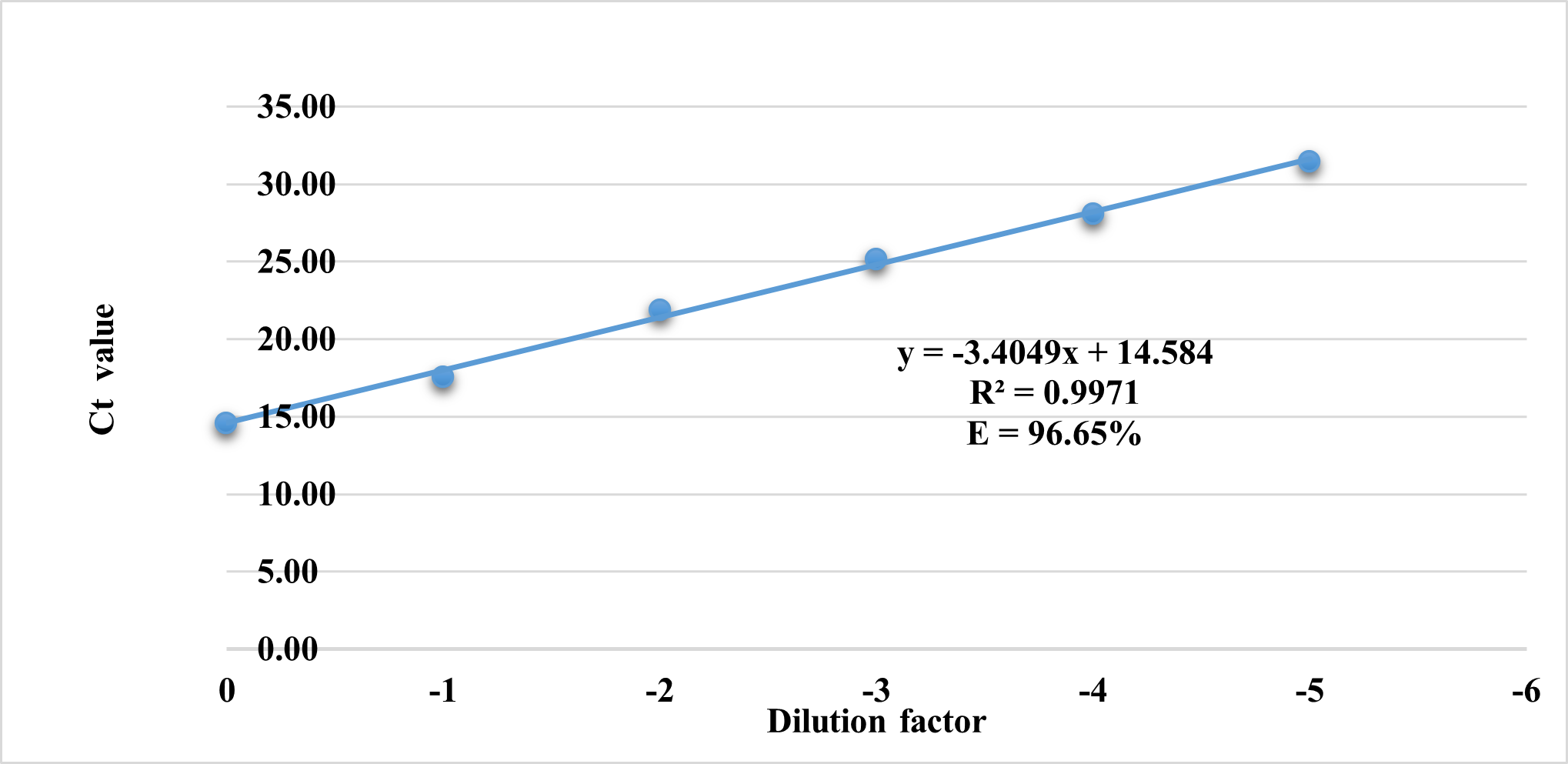
**

**Fig. S2** The efficiency of NdTryp-qPCR-F/R primers. Using the cDNA from hepatopancreas as the template, a 10-fold serial dilution was performed from the original concentration to a 1 x 10^-5^-fold dilution. The logarithmic values (Log) of these dilutions were plotted on the x-axis, while the cycle threshold (Ct) values were plotted on the y-axis. The linear regression equation derived is y = -3.4049x + 14.584, with a correlation coefficient R^2^ = 0.9971, indicating a good linear relationship. The calculated amplification efficiency (E) was 96.65%, demonstrating that the NdTryp-qPCR-F/R primer had high efficiency and accuracy across different concentrations.
