# Supplementary material for: Physiological and biochemical characterization of trypsin from Neocaridina denticulata sinensis and its roles in ontogenesis and immune response
Source: PLoS One. 2026 Feb 17;21(2):e0342746. doi: 10.1371/journal.pone.0342746 (PMC12912573; doi:10.1371/journal.pone.0342746)
Supplement: S7 File — (DOCX) [file pone.0342746.s007.docx]

**S7 File** Analysis of significant differences among different tissues.

| Tissues | Sample 1 | Sample 2 | Sample 3 | Mean | STD | Duncan |
| --- | --- | --- | --- | --- | --- | --- |
| In | 4.11 | 4.11 | 4.11 | 76533.49 | 2400.08 | a |
| Te | 59.3 | 59.3 | 59.3 | 63.68 | 4.93 | b |
| Ms | 2.41 | 2.51 | 2.68 | 20.13 | 2.06 | c |
| Ov | 15.89 | 18.13 | 26.35 | 20.12 | 5.48 | c |
| Hp | 76331.98 | 74244.68 | 79023.82 | 17.25 | 10.7 | c |
| Ep | 0.54 | 0.33 | 0.7 | 4.15 | 0.86 | d |
| Gi | 2.68 | 2.75 | 2.58 | 3.29 | 0.5 | d |
| AN | 13.74 | 29.45 | 8.57 | 2.67 | 0.09 | d |
| St | 18.64 | 19.29 | 22.47 | 2.53 | 0.14 | d |
| Ht | 3.86 | 3.12 | 2.89 | 1 | 0.07 | d |
| Es | 0.95 | 1.08 | 0.98 | 0.52 | 0.19 | d |
