# Supplementary material for: Physiological and biochemical characterization of trypsin from Neocaridina denticulata sinensis and its roles in ontogenesis and immune response
Source: PLoS One. 2026 Feb 17;21(2):e0342746. doi: 10.1371/journal.pone.0342746 (PMC12912573; doi:10.1371/journal.pone.0342746)
Supplement: Attachment — Submitted filename: 20251231_PONE-D-25-30024_R2.pdf [file pone.0342746.s017.pdf]

Reviewer’s comments

Title: Physiological and biochemical characterization of trypsin from *Neocaridina denticulata sinensis* and its roles in ontogenesis and immune response

Manuscript Number: PONE-D-25-30024R2

Journal: *PLOS ONE*

This study characterizes a trypsin gene (*NdTryp*) from the freshwater shrimp *Neocaridina denticulata sinensis* through expression profiling, developmental analysis, bacterial challenge experiments, RNAi knockdown, and biochemical characterization of a recombinant protein. However, several weaknesses limit the clarity and impact of the manuscript. The integration of developmental, immune, and biochemical roles remains largely descriptive, with mechanistic links insufficiently developed. Transitions between biological findings and proposed applications are often abrupt and speculative. In addition, despite the authors’ statement that the manuscript has undergone native English editing, **numerous sentences still contain inappropriate logical connectors, imprecise wording, and awkward sentence structures that obscure the intended arguments.** Overall, substantial refinement of sentence logic, structural coherence, and interpretative restraint is required to strengthen the manuscript. Therefore, I recommend a “**major revision**” for this manuscript in *PLOS ONE*. Specific comments are provided below:

Specific comments

Although the authors indicate that the manuscript has undergone native English editing, we and several native English–speaking colleagues still found some sentences difficult to follow due to issues in wording, sentence structure, or logical flow. The examples noted above are intended to be illustrative rather than exhaustive. In a few instances, these language issues may also reflect minor ambiguities in the underlying academic interpretation. We therefore suggest an additional round of careful revision focusing on both linguistic clarity and conceptual precision, to ensure that the study’s arguments are communicated clearly to readers.

Abstract:

The following comments highlight selected sentences with evident issues in wording, sentence structure, or logical linkage, as illustrative examples rather than an exhaustive list of all language-related concerns in this section.

1. Line 18: “Among crustaceans, trypsin is a canonical serine protease.”
2. Line 18-20: “However, in the freshwater shrimp Neocaridina denticulata sinensis, how

its contributions to ontogeny, antibacterial defense, and catalytic performance align within a single framework has not been well pinned down.”

3. Line 21-22: “Its expression was highest in the hepatopancreas, with cellular localization to storage cells (R-cells) and tubule-lining epithelial cells (ECTs).”
4. Line 22: “The signal is anatomically constrained.”
5. Line 29-30: “Taken together, these data position NdTryp as a multifunctional protease at the intersection of late embryonic development and innate antibacterial defense.”

Introduction:

The following comments highlight selected sentences with evident issues in wording, sentence structure, or logical linkage, as illustrative examples rather than an exhaustive list of all language-related concerns in this section.

1. Line 37-38: “Proteases represent one of the most abundant and diverse enzymes, responsible not only for protein degradation but also for regulating specific functional proteins through specificity to maintain the normal functioning of organisms.”
2. Lines 41–42: “Trypsin causes tissue damage due to protein hydrolysis, so trypsin is produced as an inactive precursor called trypsinogen.”
3. Lines 54–56: “A process mediated primarily by trypsin-catalyzed degradation of muscle tissue, which severely constrains shrimp shelf life during refrigerated transport.”

Materials and methods: The logical flow is acceptable; only English language editing is required.

Result: The logical flow is acceptable; only English language editing is required.

Discussion: Overall, the Discussion would be strengthened by clearer separation between data-supported conclusions and hypothesis-driven speculation, as well as by a more restrained interpretation aligned with the study’s experimental scope.
