# Supplementary material for: Physiological and biochemical characterization of trypsin from Neocaridina denticulata sinensis and its roles in ontogenesis and immune response
Source: PLoS One. 2026 Feb 17;21(2):e0342746. doi: 10.1371/journal.pone.0342746 (PMC12912573; doi:10.1371/journal.pone.0342746)
Supplement: Attachment — Submitted filename: 20260124_PONE-D-25-30024_R3.pdf [file pone.0342746.s019.pdf]

Reviewer’s comments

Title: Physiological and biochemical characterization of trypsin from *Neocaridina denticulata sinensis* and its roles in ontogenesis and immune response
Manuscript Number: PONE-D-25-30024R3
Journal: *PLOS One*

The research work entitled “Physiological and biochemical characterization of trypsin from *Neocaridina denticulata sinensis* and its roles in ontogenesis and immune response” investigates the biological functions and catalytic properties of a trypsin gene (NdTryp) in the freshwater shrimp *N. denticulata sinensis*. The authors comprehensively characterize the spatial and developmental expression patterns of NdTryp, its inducible response to *Vibrio parahaemolyticus* challenge, and the physiological consequences of RNA interference–mediated knockdown. In addition, the biochemical properties of recombinant NdTryp are systematically examined, revealing broad stability across temperature and pH ranges, as well as differential modulation by metal ions. Together, the study provides integrated physiological and biochemical evidence supporting the multifunctional roles of NdTryp in late embryonic development and innate immune defense, while also highlighting its potential application in aquafeed supplementation and enzymatic biotransformation. After three rounds of revision, the manuscript is now close to an acceptable standard, with only the following two points requiring further attention.

Specific comments

1. The phylogenetic tree lacks a scale bar indicating branch length (e.g., substitutions per site), which is necessary for interpreting evolutionary distances. Please adding this information.
2. For clarity and readability, the scale bar in Fig. 7 could be displayed at a larger scale.
