# Supplementary material for: Physiological and biochemical characterization of trypsin from Neocaridina denticulata sinensis and its roles in ontogenesis and immune response
Source: PLoS One. 2026 Feb 17;21(2):e0342746. doi: 10.1371/journal.pone.0342746 (PMC12912573; doi:10.1371/journal.pone.0342746)
Supplement: Attachment — Submitted filename: 20251103_PONE-D-25-30024 reviews comment.doc [file pone.0342746.s013.doc]

**Reviewer’s comments**

Title: Functional analysis of trypsin from *Neocaridina denticulata sinensis*: Cellular localization, ontogenesis, immune stimulation, and enzymatic characteristics

Manuscript Number: PONE-D-25-30024

Journal: *PLOS ONE*

The research work entitled “Functional analysis of trypsin from *Neocaridina denticulata sinensis*: Cellular localization, ontogenesis, immune stimulation, and enzymatic characteristics” is the first to reveal the molecular properties of trypsin in *N*. *denticulata sinensis* and its potential association with immune functions. The findings hold significant social and industrial implications: on one hand, they provide a theoretical basis for extending the shelf life of shrimp products and improving issues related to texture softening; on the other hand, understanding the role of endogenous enzymes in defense responses contributes to the development of antibiotic-free disease prevention strategies for shrimp, promoting environmentally friendly, safe, and sustainable aquaculture. However, this study still has certain shortcomings. For instance, the image resolution is too low to meet publication standards, and the overall structure of the manuscript appears somewhat disorganized, with arguments that could benefit from clearer logical flow. If the authors can improve image quality, supplement experimental data, and reorganize the section structure, the readability and academic value of the paper would be greatly enhanced. Therefore, I recommend a “**major revision**” for this manuscript in *PLOS ONE*. Specific comments are provided below:

**Specific comments**

1. The English writing of the manuscript is recommended to undergo professional language editing.
2. The title could be refined to more precisely reflect the core aspect of the study, such as its physiological characterization.
3. The current Graphical Abstract could be better aligned with the manuscript’s main theme and may benefit from being redrawn with brief explanatory text or icon labels to enhance clarity.

Abstract: The abstract is somewhat lengthy and could benefit from a clearer structure to highlight the main focus.

1. The organization of the abstract is somewhat unclear, with excessive detail that blurs the distinction between the background, objectives, methods, and conclusions. Reorganizing the structure to present these elements more concisely and clearly is recommended.

Introduction: The structure appears somewhat unclear, and the main points could be better emphasized.

1. A clearer and more focused organization in the introduction would greatly enhance overall readability. The current content appears somewhat disordered, which may hinder reader comprehension. It is recommended to condense the paragraphs and focus on presenting essential background information and the main research motivation.
2. The explanation of trypsin’s physiological roles could be streamlined, as similar descriptions appear in both the Abstract and Introduction
3. The manuscript currently addresses several broad topics—including digestive enzyme functions, antibiotic resistance, food waste utilization, and genetic engineering—which makes the overall theme somewhat diffuse. It is suggested to narrow the focus and emphasize the research motivation related to NdTryp’s physiological and immune functions, while discussing the applied aspects (e.g., food waste and enzyme recycling) later in the mauescript.

Materials and methods: Inconsistent product source labeling.

1. It is recommended to standardize the format used for indicating product sources, as some product descriptions currently lack consistency. For example: glass slides

(CITOTEST, 188105)、methanol (MACKLIN, Shanghai, China)。

Result: Revisions required.

1. The resolution of all figures is insufficient and does not meet the journal’s publication standards (https://journals.plos.org/plosone/s/figures)
2. In Fig. 1, the color markings for the poly(A) tails (901–906 bp) and the underlined region (879–900 bp) are inconsistent; additionally, the underlined region for the Trp_SPc domain does not match the description in the text (text states 236 aa, while the figure shows 232 aa)
3. In Fig. 5a, the comparison baseline is unclear. Moreover, describing *NdTryp* mRNA expression levels across different tissues as “at the same level” is inaccurate, as noticeable differences exist (e.g., in the Te tissue).
4. The logical connection and descriptive explanation between the subfigures in Fig. 6 need to be strengthened, especially for Fig. 6b.
5. The figure legends for Fig. 5, Fig. 6(a), and Fig. 9 lack explanations for the letter labels a-g shown on the graphs.
6. The legend for Table 1 is missing explanations for the symbols a-f.

Discussion: Partial revision required

1. Lines 293–294: The statement “Given that shrimp…successfully.” appears speculative and could be strengthened by adding appropriate references to support its credibility.
2. Lines 300–302: In the sentence “From the…against *V*. *parahaemolyticusit*.” the proposed concept could be clarified further by providing a clearer explanation of the underlying mechanism and supporting it with relevant references.
3. Lines 307–314: This paragraph discusses potential applications. It is recommended that the authors first complete the explanation of the trypsin-related mechanisms before introducing this section. Additionally, the logical connection between paragraphs should be strengthened to improve flow and coherence.
