# Supplementary material for: Physiological and biochemical characterization of trypsin from Neocaridina denticulata sinensis and its roles in ontogenesis and immune response
Source: PLoS One. 2026 Feb 17;21(2):e0342746. doi: 10.1371/journal.pone.0342746 (PMC12912573; doi:10.1371/journal.pone.0342746)
Supplement: Attachment — Submitted filename: Detailed Response to Reviewers.docx [file pone.0342746.s014.docx]

**Detailed Response to Reviewers and Journal Requirements**

Dear Editor,

Thank you for your useful comments and suggestions on our manuscript (**PONE-D-25-30024**). We have tried our best to revise the manuscript according to the reviewers’ suggestions, and detailed corrections are listed below point by point:

**Journal Requirements:**

When submitting your revision, we need you to address these additional requirements.

**Question 1**. Please ensure that your manuscript meets PLOS ONE's style requirements, including those for file naming. The PLOS ONE style templates can be found at

https://journals.plos.org/plosone/s/file?id=wjVg/PLOSOne_formatting_sample_main_body.pdf and

[https://journals.plos.org/plosone/s/file?id=ba62/PLOSOne_formatting_sample_title_authors_affiliations.pdf](https://track.editorialmanager.com/CL0/https:%2F%2Fjournals.plos.org%2Fplosone%2Fs%2Ffile%3Fid=ba62%2FPLOSOne_formatting_sample_title_authors_affiliations.pdf/1/010f019a6d0ca249-bd65d640-d32f-4b71-a0f9-f67a1c802eeb-000000/dCHs9YD3Mg8-gpfPMAIvE84QqALuMKJ_-JB5ZTaV3WY=236)

**Response 1.** Thank you for your email and the reminder regarding the journal’s formatting requirements. We have carefully reviewed the PLOS ONE's style templates for the main body and the title/authors/affiliations sections. We have revised our manuscript accordingly to ensure it fully complies with all the specified requirements, including file naming.

**Question 2**. Please note that funding information should not appear in any section or other areas of your manuscript. We will only publish funding information present in the Funding Statement section of the online submission form. Please remove any funding-related text from the manuscript.

**Response 2**. Any text related to funding has been removed from the revised manuscript as requested.

**Question 3**. We note that the grant information you provided in the ‘Funding Information’ and ‘Financial Disclosure’ sections do not match.

When you resubmit, please ensure that you provide the correct grant numbers for the awards you received for your study in the ‘Funding Information’ section.

**Response 3**: We have carefully reviewed and corrected the manuscript. The grant numbers in the ‘Funding Information’ section are now accurate and consistent with the ‘Financial Disclosure’ section, as you requested.

**Question 4.** Thank you for stating the following financial disclosure:

“This work was supported by the National Natural Science Foundation of China (Grant Nos. 32172954, 32373121) and the Natural Science Foundation of Hebei Province of China (D2023201002).”

Please state what role the funders took in the study. If the funders had no role, please state: "The funders had no role in study design, data collection and analysis, decision to publish, or preparation of the manuscript."

If this statement is not correct you must amend it as needed.

Please include this amended Role of Funder statement in your cover letter; we will change the online submission form on your behalf.

**Response 4**: As requested, we have now added the role of the funders to the ‘Funding’ section of our revised manuscript. The amended statement is as follows: The funders (Jiquan Zhang) had a role in the study design, data collection, and analysis, as well as the decision to publish. We have also included this statement in our revised cover letter.

**Question 5.** In the online submission form you indicate that your data is not available for proprietary reasons and have provided a contact point for accessing this data. Please note that your current contact point is a co-author on this manuscript. According to our Data Policy, the contact point must not be an author on the manuscript and must be an institutional contact, ideally not an individual. Please revise your data statement to a non-author institutional point of contact, such as a data access or ethics committee, and send this to us via return email. Please also include contact information for the third party organization, and please include the full citation of where the data can be found.

**Response 5**: As our data is part of an ongoing study, it cannot be deposited in a public repository at this time. However, according to your instructions, we have included all the basic data of this study as supporting information files.

**Question 6**. PLOS requires an ORCID iD for the corresponding author in Editorial Manager on papers submitted after December 6th, 2016. Please ensure that you have an ORCID iD and that it is validated in Editorial Manager. To do this, go to ‘Update my Information’ (in the upper left-hand corner of the main menu), and click on the Fetch/Validate link next to the ORCID field. This will take you to the ORCID site and allow you to create a new iD or authenticate a pre-existing iD in Editorial Manager.

**Response 6:** Thank you for reminding us about the ORCID iD requirement. I have now validated my ORCID iD in the Editorial Manager system as requested. The ORCID iD (0000-0001-5063-339x) has been successfully linked to my account through the ‘Update my Information’ section.

**Question 7.** Your ethics statement should only appear in the Methods section of your manuscript. If your ethics statement is written in any section besides the Methods, please move it to the Methods section and delete it from any other section. Please ensure that your ethics statement is included in your manuscript, as the ethics statement entered into the online submission form will not be published alongside your manuscript.

**Response 7:** Thank you for your guidance regarding the ethics statement placement. Following your instructions, we have reviewed our manuscript and confirmed that the ethics statement now appears only in the Materials and methods section (Section 2.1). In addition, we have removed any duplicate ethics statements from other sections of the manuscript.

**Question 8.** Please include captions for your Supporting Information files at the end of your manuscript, and update any in-text citations to match accordingly. Please see our Supporting Information guidelines for more information: [http://journals.plos.org/plosone/s/supporting-information](http://track.editorialmanager.com/CL0/http:%2F%2Fjournals.plos.org%2Fplosone%2Fs%2Fsupporting-information/1/010f019a6d0ca249-bd65d640-d32f-4b71-a0f9-f67a1c802eeb-000000/Uxvq9-WoZPDQDjn-7eqHH1UHkG-ifEal7ROTZH79VW8=236).

**Response 8:** Thank you for your guidance regarding the Supporting Information files. Following your instructions, we have now included captions for all Supporting Information files at the end of our manuscript. We have also updated all in-text citations to ensure they match the Supporting Information captions accordingly.

**Question 9.** PLOS ONE now requires that authors provide the original uncropped and unadjusted images underlying all blot or gel results reported in a submission’s figures or Supporting Information files. This policy and the journal’s other requirements for blot/gel reporting and figure preparation are described in detail at https://journals.plos.org/plosone/s/figures#loc-blot-and-gel-reporting-requirements and https://journals.plos.org/plosone/s/figures#loc-preparing-figures-from-image-files. When you submit your revised manuscript, please ensure that your figures adhere fully to these guidelines and provide the original underlying images for all blot or gel data reported in your submission. See the following link for instructions on providing the original image data: https://journals.plos.org/plosone/s/figures#loc-original-images-for-blots-and-gels.

In your cover letter, please note whether your blot/gel image data are in Supporting Information or posted at a public data repository, provide the repository URL if relevant, and provide specific details as to which raw blot/gel images, if any, are not available. Email us at [plosone@plos.org](mailto:plosone@plos.org) if you have any questions.

**Response 9:** Thank you for your guidance regarding the original image requirements for blot and gel data. We have carefully reviewed the journal’s guidelines and have provided the original, unadjusted underlying image for the SDS-PAGE gel reported in **Fig. 8**. This raw image has been included as a Supporting Information file (S8 File), and a corresponding caption has been added at the end of the manuscript as requested. The original full-size gel also contained lanes from a separate, unrelated study, and was therefore physically cut before imaging. The image we have provided is the complete and unadjusted original for all lanes pertaining to this manuscript.

**Question 10.** If the reviewer comments include a recommendation to cite specific previously published works, please review and evaluate these publications to determine whether they are relevant and should be cited. There is no requirement to cite these works unless the editor has indicated otherwise.

**Response 10:** We sincerely thank the editor for this insightful and constructive comment. We have carefully reviewed and evaluated the cited references to determine their relevance to this study.

**Reviewers’ comments**

**Reviewer #1, Comments to PONE-D-25-30024:**

**Question 1.** Data availability does not comply with PLOS ONE policy.

Your current statement that “data are available upon reasonable request” is not sufficient. All underlying data should be made publicly accessible via a repository or included in the manuscript/Supplementary Information. Please revise accordingly.

**Response 1:** Thank you for your guidance regarding the Data Availability Statement. We have revised the manuscript accordingly. All underlying data have now been included as Supporting Information files, and the Data Availability Statement has been updated to reflect this change.

**Question 2**. English usage requires substantial improvement.
The manuscript contains numerous instances of repetitive or awkward sentence structures, including excessive passive voice. A thorough language editing by a native English speaker or a professional service is strongly advised.

**Response 2:** Thanks for the reviewer’s advice. We read the paper carefully and invited an Indian professor to help modify the language.

**Question 3.** Statistical details in figures are insufficient.

Figures such as Fig. 5 and Fig. 6 lack proper annotation of statistical significance (e.g., a/b/c labelling, p-values). Please include exact sample sizes (n), error bars (mean ± SD or SEM), and statistical tests used in each figure legend.

**Response 3:** We sincerely thank the reviewer for constructive comments. We have annotated the statistical significance in the legends of the figures. The exact sample size (*n*), error bars (mean ± SD), and statistical tests were added in the Materials and methods section.

**Question 4**. Visual presentation of figures requires enhancement.

Images in Fig. 6 and Fig. 7 (H&E and ISH) lack scale bars and cellular annotations. Please ensure all micrographs are labelled clearly with appropriate magnification and cell types.

**Response 4:** Thank you for your valuable feedback regarding the visual presentation of our figures. The issue was caused by the submission system’s file size limitations, which required us to compress the images. This, unfortunately, resulted in a loss of detail, making the scale bars and cellular annotations difficult to read. Our original, high-resolution micrographs do contain clear scale bars and annotations. To address this, we have taken the following steps: We have uploaded the original, high-resolution versions of **Fig. 6** and **Fig. 7** as a new Supporting Information file named ‘S4-S7 File: High resolution **Fig. 6** and **Fig. 7**’. These images clearly show all the required scale bars and cellular annotations (e.g., B-cells, R-cells, ECTs).

**Question** 5. Potential mechanistic insight is underdeveloped.

While you report that NdTryp may be involved in immune defence against Vibrio parahaemolyticus, the molecular mechanism remains speculative. Please elaborate on possible pathways or propose a model, even if hypothetical.

**Response 5:** We sincerely thank the reviewer for this insightful and constructive comment. Based on your suggestion, we have added a detailed explanation and hypothetical model of the immune pathways that NdTryp may be involved in in the Discussion section of the revised manuscript to provide deeper mechanistic insights. Please refer to page 11, lines 311-315 of the revised manuscript for details.

**Question 6**. Discussion contains overextended claims.

Statements regarding industrial application for food waste degradation or environmental protection should be toned down or qualified, as current evidence is preliminary.

**Response 6:** We sincerely thank the reviewer for this insightful and constructive comment. Based on the reviewer’s suggestion, we have carefully revised the Discussion and Conclusions sections, weakening and limiting the relevant statements to make them more rigorous and in line with existing evidence. Please refer to page 12, lines 316-326, and page 13, lines 347-350 of the revised manuscript for details.

**Question 7:** Ethical statements are acceptable but could be clarified.

Although no vertebrate animals were used, please state explicitly whether all procedures were approved by institutional guidelines and whether ARRIVE guidelines were followed.

**Response 7:** Thank you for your feedback and the suggestion to clarify our ethical statement. We agree that a more explicit statement enhances the transparency of our work. Following your recommendation, we have revised and consolidated the ethical statement in the Methods section of our manuscript. The revised statement now explicitly confirms that all procedures were approved by institutional guidelines and that the ARRIVE guidelines were followed. To improve clarity, we have revised the relevant section in the manuscript (Section 2.1, pages 3-4) to explicitly state these points.

**Question 8:** Literature references need updating and better integration.
Some cited references are outdated (e.g., 1990s), and others are not effectively linked to claims made in the text. Please ensure recent and relevant studies are cited appropriately.

**Response 8:** Thank you for your valuable comment regarding the literature references in our manuscript. We acknowledge that some references from the 1990s are cited in our manuscript. However, we would like to respectfully clarify that these older references represent seminal and foundational works in the field of crustacean trypsin research. In addition, we conducted a comprehensive search and review of recent literature. The literature with strong relevance has been added to the revised manuscript, while the literature with low relevance has been removed from the citation list. Please refer to the References section for detailed information.

**Question** 9. Clarify biological replicates and experimental repeats.

While some methods mention “three replicates,” it is unclear whether these are technical or biological replicates. Please clarify replication strategy for each major experiment.

**Response 9:** Thank you for your valuable feedback regarding the clarification of our experimental replicates. We have now revised the Materials and Methods section to explicitly differentiate between biological and technical replicates for each major experiment. Please refer to page 5, line 121, lines 129-130, lines 140-141; and page 7, lines 198-200 of the revised manuscript for details.

**Reviewers’ comments**

**Reviewer #2, Comments to PONE-D-25-30024:**

Reviewer #2: The research work entitled “Functional analysis of trypsin from Neocaridina denticulata sinensis: Cellular localization, ontogenesis, immune stimulation, and enzymatic characteristics” is the first to reveal the molecular properties of trypsin in *N. denticulata sinensis* and its potential association with immune functions. The findings hold significant social and industrial implications: on one hand, they provide a theoretical basis for extending the shelf life of shrimp products and improving issues related to texture softening; on the other hand, understanding the role of endogenous enzymes in defense responses contributes to the development of antibiotic-free disease prevention strategies for shrimp, promoting environmentally friendly, safe, and sustainable aquaculture. However, this study still has certain shortcomings. For instance, the image resolution is too low to meet publication standards, and the overall structure of the manuscript appears somewhat disorganized, with arguments that could benefit from clearer logical flow. If the authors can improve image quality, supplement experimental data, and reorganize the section structure, the readability and academic value of the paper would be greatly enhanced. Therefore, I recommend a “major revision” for this manuscript in PLOS ONE. Specific comments are provided below:

Specific comments

**Question 1.** The English writing of the manuscript is recommended to undergo professional language editing.

**Response 1:** Thanks for the reviewer’s advice. We read the paper carefully and invited an Indian professor to help modify the language.

**Question 2.** The title could be refined to more precisely reflect the core aspect of the study, such as its physiological characterization.

**Response 2:** Thank you very much for the valuable suggestions provided by the reviewer. We agree with your opinion that the original title can indeed be more refined and accurate in highlighting the physiological characteristics of the research core. In order to better summarize the contribution of this study, we have revised the title. The title of the revised manuscript is: Physiological and biochemical characterization of trypsin from *Neocaridina denticulata sinensis* and its roles in ontogenesis and immune response.

**Question 3**. The current Graphical Abstract could be better aligned with the manuscript’s main theme and may benefit from being redrawn with brief explanatory text or icon labels to enhance clarity.

**Response 3:** Thank you very much for the valuable suggestions provided by the reviewer. We agree that the original graphic abstract needs improvement in highlighting the research topic and clarity. Based on your suggestion, we have redesigned and optimized the graphical abstract.

Abstract: The abstract is somewhat lengthy and could benefit from a clearer structure to highlight the main focus.

**Question 1.** The organization of the abstract is somewhat unclear, with excessive detail that blurs the distinction between the background, objectives, methods, and conclusions. Reorganizing the structure to present these elements more concisely and clearly is recommended.

**Response 1:** Thanks for the reviewer’s advice. According to your suggestion, we have thoroughly rewritten and reorganized the abstract to make its structure clearer and its content more concise, enabling a clear distinction among background, methods, results, and conclusions.

Introduction: The structure appears somewhat unclear, and the main points could be better emphasized.

**Question 1.** A clearer and more focused organization in the introduction would greatly enhance overall readability. The current content appears somewhat disordered, which may hinder reader comprehension. It is recommended to condense the paragraphs and focus on presenting essential background information and the main research motivation.

**Response 1:** Thank you very much for the valuable suggestions provided by the reviewer. We have substantially revised and reorganized the Introduction section to improve its clarity and focus. Please refer to pages 2-3, lines 35-58 of the revised manuscript for details.

**Question 2.** The explanation of trypsin’s physiological roles could be streamlined, as similar descriptions appear in both the Abstract and Introduction

**Response 2:** Thank you very much for the valuable suggestions provided by the reviewer. We have simplified the description of the physiological role of trypsin in the revised manuscript. In addition, to avoid duplication with the Abstract content, we have rewritten the Abstract section.

**Question 3.** The manuscript currently addresses several broad topics—including digestive enzyme functions, antibiotic resistance, food waste utilization, and genetic engineering—which makes the overall theme somewhat diffuse. It is suggested to narrow the focus and emphasize the research motivation related to NdTryp’s physiological and immune functions, while discussing the applied aspects (e.g., food waste and enzyme recycling) later in the mauescript.

**Response 3:** Thank you very much for the valuable suggestions provided by the reviewer. We have followed the requirements to narrow down the scope of our research. In the revised manuscript, we mainly emphasize the research motivation related to the physiology and immune function of NdTryp. The paragraph regarding food waste has been deleted and will only discuss the application of food waste and enzyme recycling in the later manuscript. Please refer to page 11, lines 309-326 of the revised manuscript for details.

Materials and methods: Inconsistent product source labeling.

**Question 1.** It is recommended to standardize the format used for indicating product sources, as some product descriptions currently lack consistency. For example: glass slides

(CITOTEST, 188105)、methanol (MACKLIN, Shanghai, China).

**Response 1:** Thank you very much for the valuable suggestions provided by the reviewer. We have carefully reviewed the entire text and standardized the description format for all reagents, consumables, and test kits as follows: product name (company name, city, country).

Result: Revisions required.

**Question 1.** The resolution of all figures is insufficient and does not meet the journal’s publication standards (<https://journals.plos.org/plosone/s/figures>)

**Response 1:** Thank you very much for the valuable suggestions provided by the reviewer. In fact, we have already uploaded all original high-resolution figures (**Fig. 1-9**) as separate files to the submission system according to the journal’s requirements. These original figure files are quality and fully comply with the journal’s resolution requirements (e.g., 300 DPI or higher). You should be able to view or download the original high-resolution figures by clicking on the figure links (the arrow) in the review system of the PDF file.

**Question 2.** In Fig. 1, the color markings for the poly(A) tails (901–906 bp) and the underlined region (879–900 bp) are inconsistent; additionally, the underlined region for the Trp_SPc domain does not match the description in the text (text states 236 aa, while the figure shows 232 aa)

**Response 2:** Thank you very much for the valuable suggestions provided by the reviewer. we have thoroughly re-examined both the figure and the text. We performed a new domain prediction analysis using the SMART online tool, and the results revealed that the Tryp_SPc domain actually contains 233 amino acids (rather than the 236 aa stated in the original text or the 232 aa shown in the original figure). In addition, we have redrawn **Fig. 1** to ensure that the marking for the poly(A) tail (red double underline) accurately corresponds to its position in the sequence (901–906 bp), thus eliminating the previous ambiguity.

**Question 3.** In Fig. 5a, the comparison baseline is unclear. Moreover, describing NdTryp mRNA expression levels across different tissues as “at the same level” is inaccurate, as noticeable differences exist (e.g., in the Te tissue).

**Response 3:** We sincerely thank the reviewer for this insightful and constructive comment. We reanalyzed the data and modified the description of **Fig. 5a.** To convey our findings more precisely and prevent any possible misinterpretation, we have revised the corresponding description in the Results section accordingly. For specific details, please refer to lines 232-235 on pages 8-9 of the revised manuscript.

**Question 4.** The logical connection and descriptive explanation between the subfigures in Fig. 6 need to be strengthened, especially for Fig. 6b.

**Response 4:** We sincerely thank the reviewer for this insightful and constructive comment. We designed the experiments in **Fig. 6** with the following logic:

**1)** We observed that the *NdTryp* gene expression was significantly upregulated following a *V. parahaemolyticus* challenge (**Fig. 6a**), suggesting its involvement in the immune response. To test this, we used RNAi to assess the function of NdTryp. As a critical preliminary step, we first determined the optimal time for achieving maximum gene knockdown.

**2) Fig. 6b** shows that the most significant suppression of *NdTryp* expression occurred at 24 h post-injection of dsRNA. Consequently, we used this 24-hour time point for our subsequent functional challenge.

**3)** The results, shown in **Fig. 6c** and **Fig. 6d**, revealed that while the control group’s hepatopancreas remained intact (**Fig. 6c**), the *NdTryp*-knockdown shrimp suffered severe tissue damage and necrosis after being challenged with the pathogen (**Fig. 6d**). This sequence of experiments provides strong evidence that NdTryp is crucial for host defense against bacterial infection and for maintaining the structural integrity of the hepatopancreas.

To clarify this logic, we have revised the descriptions in both the Results (Section 3.3) and the **Fig. 6** legend to better connect the subfigures and explain their purpose.

**Question 5.** The figure legends for Fig. 5, Fig. 6(a), and Fig. 9 lack explanations for the letter labels a-g shown on the graphs.

**Response 5:** We sincerely thank the reviewer for this insightful and constructive comment. We have explained the letter labels displayed on **Fig. 5**, **Fig. 6a**, and **Fig. 9** in the legend section of the revised manuscript.

**Question 6.** The legend for Table 1 is missing explanations for the symbols a-f.

**Response 6:** We sincerely thank the reviewer for this insightful and constructive comment. In the revised manuscript, we have added the following explanation to the Table 1 legend: “Values with different lowercase letters (a, b, c, d, e, f) are significantly different among treatment groups (*p* < 0.05). Values sharing a common letter are not significantly different (*p* > 0.05).” This clarification now properly explains the statistical significance of the letter designations used in the table.

Discussion: Partial revision required

**Question 1.** Lines 293–294: The statement “Given that shrimp…successfully.” appears speculative and could be strengthened by adding appropriate references to support its credibility.

**Response 1:** Thank you very much for the valuable suggestions provided by the reviewer. We have added relevant references in the revised manuscript. For specific details, please refer to line 301 on page 11 of the revised manuscript.

**Question 2.** Lines 300–302: In the sentence “From the…against V. parahaemolyticusit.” the proposed concept could be clarified further by providing a clearer explanation of the underlying mechanism and supporting it with relevant references.

**Response 2:** Thank you very much for the valuable suggestions provided by the reviewer. We have rewritten this sentence and added relevant references to provide a more detailed explanation and clarification of this section. Please refer to lines 311-315 on page 11 of the revised manuscript for specific details.

**Question 3.** Lines 307–314: This paragraph discusses potential applications. It is recommended that the authors first complete the explanation of the trypsin-related mechanisms before introducing this section. Additionally, the logical connection between paragraphs should be strengthened to improve flow and coherence.

**Response 3:** Thank you very much for the valuable suggestions provided by the reviewer. We have supplemented the explanation of trypsin-related mechanisms based on the reviewer’s comments. At the same time, we have also strengthened the logical connections between paragraphs to enhance the fluency and coherence of the revised manuscript. For specific details, please refer to lines 311-326 on page 11 of the revised manuscript.
