# Supplementary material for: Physiological and biochemical characterization of trypsin from Neocaridina denticulata sinensis and its roles in ontogenesis and immune response
Source: PLoS One. 2026 Feb 17;21(2):e0342746. doi: 10.1371/journal.pone.0342746 (PMC12912573; doi:10.1371/journal.pone.0342746)
Supplement: Attachment — Submitted filename: 20251202_PONE-D-25-30024_R1.doc [file pone.0342746.s015.doc]

**Reviewer’s comments**

Title: Physiological and biochemical characterization of trypsin from *Neocaridina denticulata sinensis* and its roles in ontogenesis and immune response

Manuscript Number: PONE-D-25-30024R1

Journal: *PLOS ONE*

The research work entitled “Physiological and biochemical characterization of trypsin from *Neocaridina denticulata sinensis* and its roles in ontogenesis and immune response”

presents interesting data on the characterization of NdTryp and addresses a topic of potential relevance to crustacean physiology and immunity. However, several aspects of the writing and organization currently limit the clarity and overall impact of the work. Across multiple sections, the narrative tends to lose focus, with frequent inclusion of background information or speculative interpretations that extend beyond the scope of the presented data. As a result, the central findings are sometimes overshadowed, and the connection between the study’s aims, methods, results, and broader significance becomes less clear. Substantial revisions to the structure, conciseness, and alignment of each section with the core objectives of the study would greatly improve readability and help strengthen the manuscript’s scientific message. In addition, several sentences would benefit from careful English editing to enhance clarity, precision, and overall fluency. Therefore, I recommend a “**minor revision**” for this manuscript in *PLOS ONE*. Specific comments are provided below:

**Specific comments**

This manuscript remains linguistically unqualified for publication. It is strongly recommended to have the entire text thoroughly revised by a professional native English editor.

Abstract:

1. The abstract reads as a list of results rather than a cohesive summary. The study rationale and knowledge gap are insufficiently stated.
2. Several experimental details (e.g., specific metal ions) are too fine-grained for an abstract and should be summarized.
3. The logical flow is fragmented; developmental data, immune response, and biochemical assays appear disconnected.

Suggestions for Restructuring:

1. Begin with a concise background that clearly states the research gap.
2. Follow with a single sentence summarizing the study aim.
3. Group major findings into biological (expression/immune) and biochemical (activity) results.
4. End with a clear, complete significance statement relevant to crustacean biology or aquaculture.

Introduction: A more concise and structured presentation will better guide readers toward the specific aims of the study.

1. Certain sections provide extensive textbook-level descriptions and broader contextual information (e.g., food waste, CO₂ emissions, industrial proteases) that may not directly support the central theme of the study. Streamlining these areas would help maintain focus.
2. Background information relevant to this species appears somewhat late in the Introduction and might be more effective if moved forward.
3. The knowledge gap is not clearly stated. It is unclear what is unknown about trypsin in *N*. *denticulata sinensis*, and how this study fills that gap.

Materials and methods: Partial revision required

1. The distinction between biological and technical replicates could be described more clearly across experiments.
2. The reported dsRNA concentration seems unusually high (1,000 μg/μL); confirming this value would strengthen confidence in the RNAi experiments.

Result: The logical flow is acceptable; only English language editing is required.

Discussion: Partial revision required

A more targeted discussion will help emphasize the contributions of this study and align them closely with the presented data.

1. The flow of ideas occasionally shifts between distant topics, and organizing the discussion around the key results (developmental expression, immune response, biochemical properties) may improve clarity.
2. Certain important findings, such as the expression pattern and RNAi phenotypes, could be discussed in greater depth with regard to their biological relevance.
