# Supplementary material for: Physiological and biochemical characterization of trypsin from Neocaridina denticulata sinensis and its roles in ontogenesis and immune response
Source: PLoS One. 2026 Feb 17;21(2):e0342746. doi: 10.1371/journal.pone.0342746 (PMC12912573; doi:10.1371/journal.pone.0342746)
Supplement: Attachment — Submitted filename: Detailed_Response_to_Reviewers_auresp_2.docx [file pone.0342746.s016.docx]

**Detailed Response to Reviewers**

Dear Editor,

Thank you for your useful comments and suggestions on our manuscript (**PONE-D-25-30024R1**). We have tried our best to revise the manuscript according to the reviewers’ suggestions, and detailed corrections are listed below point by point:

**Reviewers’ comments**

**Reviewer #1**: This manuscript has been fully revised in accordance with the reviewers’ comments and is now suitable for publication.

**Response to Reviewer #1:** We are very pleased to learn that you are satisfied with our revisions and agree that all previous concerns have been addressed. We are sincerely grateful for your time and effort in this process. Your insightful and constructive feedback has been invaluable and has significantly improved the quality and clarity of our manuscript.

**Reviewers’ comments**

Title: Physiological and biochemical characterization of trypsin from *Neocaridina denticulata sinensis* and its roles in ontogenesis and immune response

Manuscript Number: PONE-D-25-30024R1

Journal: *PLOS ONE*

The research work entitled “Physiological and biochemical characterization of trypsin from *Neocaridina denticulata sinensis* and its roles in ontogenesis and immune response” presents interesting data on the characterization of NdTryp and addresses a topic of potential relevance to crustacean physiology and immunity. However, several aspects of the writing and organization currently limit the clarity and overall impact of the work. Across multiple sections, the narrative tends to lose focus, with frequent inclusion of background information or speculative interpretations that extend beyond the scope of the presented data. As a result, the central findings are sometimes overshadowed, and the connection between the study’s aims, methods, results, and broader significance becomes less clear. Substantial revisions to the structure, conciseness, and alignment of each section with the core objectives of the study would greatly improve readability and help strengthen the manuscript’s scientific message. In addition, several sentences would benefit from careful English editing to enhance clarity, precision, and overall fluency. Therefore, I recommend a “**minor revision**” for this manuscript in *PLOS ONE*. Specific comments are provided below:

**Question:** Specific comments

This manuscript remains linguistically unqualified for publication. It is strongly recommended to have the entire text thoroughly revised by a professional native English editor.

**Response:** Thank you for your comment. We fully agree that the English language needs substantial improvement. The entire manuscript (including the abstract and main text) has now been thoroughly revised by a professional native English editor to improve grammar, clarity, readability, and academic style. We have also carefully checked terminology consistency throughout the manuscript.

**Abstract**:

**Question 1**. The abstract reads as a list of results rather than a cohesive summary. The study rationale and knowledge gap are insufficiently stated.

**Response 1:** We thank the reviewer for this valuable suggestion. We have further refined the abstract to enhance its clarity, logical flow, and scientific rigor while maintaining its comprehensive coverage of our key findings. The modifications specifically address:

**1)** improved logical progression from background to objectives to results.

**2)** enhanced quantitative precision in reporting key findings.

**3)** strengthened emphasis on the novelty and implications of our work.

**4)** Better integration of functional and biotechnological aspects.

We believe these refinements have strengthened the manuscript’s presentation and accessibility to a broader readership.

**Question 2**. Several experimental details (e.g., specific metal ions) are too fine-grained for an abstract and should be summarized.

**Response 2:** We thank the reviewer for this valuable suggestion. Therefore, we revised the Abstract by:

**1)** removing the enumeration of individual ions and associated quantitative effects.

**2)** summarizing this section as “activity was modulated by divalent/trivalent metal ions,” while retaining the key take-home message that rNdTryp shows broad pH/temperature tolerance and ion-dependent modulation.

**Question 3**. The logical flow is fragmented; developmental data, immune response, and biochemical assays appear disconnected.

Suggestions for Restructuring:

1. Begin with a concise background that clearly states the research gap.

2. Follow with a single sentence summarizing the study aim.

3. Group major findings into biological (expression/immune) and biochemical (activity) results.

4. End with a clear, complete significance statement relevant to crustacean biology or aquaculture.

**Response 3:** We thank the reviewer for this insightful suggestion. We have revised the Abstract to improve coherence and ensure a single, continuous logic chain.

**1)** The background/research gap was moved to the opening sentence to clarify what is unknown regarding NdTryp across development, immunity, and enzymatic traits.

**2)** We added one explicit aim sentence to unify the experimental modules (gene characterization, biological function, and recombinant biochemical profiling).

**3)** We grouped the major findings into two clearly labeled blocks.

**4)** We rewrote the final sentence as a complete significance statement highlighting relevance to crustacean biology and aquaculture/biotechnology potential.

**Introduction**: A more concise and structured presentation will better guide readers toward the specific aims of the study.

**Question 1**. Certain sections provide extensive textbook-level descriptions and broader contextual information (e.g., food waste, CO₂ emissions, industrial proteases) that may not directly support the central theme of the study. Streamlining these areas would help maintain focus.

**Response 1:** Thank you for your feedback and the suggestion. The corresponding parts of the manuscript have been rewritten, removing extensive textbook-level descriptions and broader contextual information such as food waste, CO₂ emissions, and industrial proteases.

**Question 2**. Background information relevant to this species appears somewhat late in the Introduction and might be more effective if moved forward.

**Response 2:** Thank you for this helpful suggestion. We have revised the Introduction by moving the background information relevant to this species to an earlier position and updating the relevant references accordingly, so that the rationale and context are established upfront (see revised **Introduction** section).

**Question 3**. The knowledge gap is not clearly stated. It is unclear what is unknown about trypsin in *N. denticulata sinensis*, and how this study fills that gap.

**Response 3:** We sincerely thank the reviewer for this constructive comment. We agree that the knowledge gap in the original manuscript was insufficiently articulated. To address this concern, we have substantially revised the Introduction to explicitly state what remains unknown about trypsin in *Neocaridina denticulata sinensis* and how our study systematically fills these gaps. Please refer to page 3, lines 64-67 of the revised manuscript for details.

**Materials and methods**: Partial revision required

**Question 1**. The distinction between biological and technical replicates could be described more clearly across experiments.

**Response 1:** We thank the reviewer for raising this important point regarding experimental replicates. We have carefully reviewed our manuscript and provided clarifications as follows:

**1. Biological Replicates**

- For tissue expression analysis (Section 2.6)
- For bacterial challenge experiments (Section 2.5)
- For the embryonic development study (Section 2.6)

**2. Technical Replicates**

- For qPCR assays (Section 2.6)
- For enzymatic characterization (Section 2.10)

**3. Statistical Analysis Clarification**

- Statistical analysis (Section 2.11)

**Question 2**. The reported dsRNA concentration seems unusually high (1,000 μg/μL); confirming this value would strengthen confidence in the RNAi experiments.

**Response 2:** Thank you for pointing this out. The dsRNA concentration “1,000 μg/μL” was a Unit typographical error. The correct concentration is 1,000 ng/μL (i.e., 1 μg/μL). We have corrected the unit throughout the manuscript (**2.4 Methodology of RNA interference (RNAi)** section, page 5, lines 113–114). This correction does not affect the RNAi experimental design, dosage, or the conclusions of the study.

**Question** Result: The logical flow is acceptable; only English language editing is required.

**Response:** Thank you for this helpful suggestion. We have carefully revised the English language throughout the manuscript with the assistance of a native English-speaking colleague. The revisions addressed grammar, sentence clarity, and consistency in scientific expression. We believe the revised manuscript now meets the journal’s language standards.

**Discussion**: Partial revision required

A more targeted discussion will help emphasize the contributions of this study and align them closely with the presented data.

**Question 1**. The flow of ideas occasionally shifts between distant topics, and organizing the discussion around the key results (developmental expression, immune response, biochemical properties) may improve clarity.

**Response 1:** Thank you for this constructive suggestion regarding the organization and flow of the Discussion section. To improve the logical structure, our key findings would enhance manuscript clarity. We have completely reorganized the Discussion section into four clearly delineated subsections:

**1) Section 4.1 - Structural features and evolutionary conservation of NdTryp**: We now begin with the molecular foundation, discussing the catalytic triad, calcium-binding sites, and β-barrel architecture that underpin biochemical properties of NdTryp.

**2) Section 4.2 - NdTryp expression and its biological significance**: This section provides a focused analysis of the developmental expression pattern, interpreting the biphasic profile in the context of yolk dependence, exogenous feeding transition, and potential hatching facilitation.

**3) Section 4.3 - Proposed immune mechanisms**: We consolidated all immune-related findings here, including:

- Expression dynamics following *V. parahaemolyticus* challenge;
- RNAi phenotype description and interpretation;
- Proposed mechanistic pathways;
- Integration of histopathological observations with molecular mechanisms.

**4) Section 4.4 - Biochemical properties and biotechnological potential**: This section synthesizes enzymatic characterization data with practical applications, concluding with cellular localization findings and production feasibility considerations.

**Question 2**. Certain important findings, such as the expression pattern and RNAi phenotypes, could be discussed in greater depth with regard to their biological relevance.

**Response 2:** Thank you for your insightful comment regarding the biological relevance of NdTryp expression patterns and RNAi phenotypes. We agree that these important findings warrant deeper discussion, and we have substantially revised the Discussion section accordingly and removed extraneous statements.

**1) Regarding the high expression of *NdTryp* in hepatopancreas:**

We have expanded our discussion to address the biological significance of the exceptionally high *NdTryp* expression in hepatopancreas compared to other tissues (Page 11, Lines 299-304).

**2) Regarding the RNAi-induced hepatopancreatic necrosis:**

We have added the mechanistic discussion explaining how *NdTryp* knockdown leads to severe tissue necrosis (Pages 11-12, Lines 314-338).
