# Supplementary material for: Physiological and biochemical characterization of trypsin from Neocaridina denticulata sinensis and its roles in ontogenesis and immune response
Source: PLoS One. 2026 Feb 17;21(2):e0342746. doi: 10.1371/journal.pone.0342746 (PMC12912573; doi:10.1371/journal.pone.0342746)
Supplement: Attachment — Submitted filename: Detailed_Response_to_Reviewers_auresp_3.docx [file pone.0342746.s018.docx]

**Detailed Response to Reviewers**

Dear Editor,

Thank you for your useful comments and suggestions on our manuscript (**PONE-D-25-30024R2**). We have tried our best to revise the manuscript according to the reviewers’ suggestions, and detailed corrections are listed below point by point:

Reviewer #2: Reviewer’s comments

Title: Physiological and biochemical characterization of trypsin from *Neocaridina denticulata sinensis* and its roles in ontogenesis and immune response

Manuscript Number: PONE-D-25-30024R2

Journal: *PLOS ONE*

This study characterizes a trypsin gene (*NdTryp*) from the freshwater shrimp *Neocaridina denticulata sinensis* through expression profiling, developmental analysis, bacterial challenge experiments, RNAi knockdown, and biochemical characterization of a recombinant protein. However, several weaknesses limit the clarity and impact of the manuscript. The integration of developmental, immune, and biochemical roles remains largely descriptive, with mechanistic links insufficiently developed. Transitions between biological findings and proposed applications are often abrupt and speculative. In addition, despite the authors’ statement that the manuscript has undergone native English editing, **numerous sentences still contain inappropriate logical connectors, imprecise wording, and awkward sentence structures that obscure the intended arguments**. Overall, substantial refinement of sentence logic, structural coherence, and interpretative restraint is required to strengthen the manuscript. Therefore, I recommend a “**major revision**” for this manuscript in *PLOS ONE*. Specific comments are provided below:

Specific comments

Although the authors indicate that the manuscript has undergone native English editing, we and several native English–speaking colleagues still found some sentences difficult to follow due to issues in wording, sentence structure, or logical flow. The examples noted above are intended to be illustrative rather than exhaustive. In a few instances, these language issues may also reflect minor ambiguities in the underlying academic interpretation. We therefore suggest an additional round of careful revision focusing on both linguistic clarity and conceptual precision, to ensure that the study’s arguments are communicated clearly to readers.

Abstract:

The following comments highlight selected sentences with evident issues in wording, sentence structure, or logical linkage, as illustrative examples rather than an exhaustive list of all language-related concerns in this section.

1. Line 18: “Among crustaceans, trypsin is a canonical serine protease.”

2. Line 18-20: “However, in the freshwater shrimp *Neocaridina denticulata sinensis*, how its contributions to ontogeny, antibacterial defense, and catalytic performance align within a single framework has not been well pinned down.”

3. Line 21-22: “Its expression was highest in the hepatopancreas, with cellular localization to storage cells (R-cells) and tubule-lining epithelial cells (ECTs).”

4. Line 22: “The signal is anatomically constrained.”

5. Line 29-30: “Taken together, these data position NdTryp as a multifunctional protease at the intersection of late embryonic development and innate antibacterial defense.”

Introduction:

The following comments highlight selected sentences with evident issues in wording, sentence structure, or logical linkage, as illustrative examples rather than an exhaustive list of all language-related concerns in this section.

1. Line 37-38: “Proteases represent one of the most abundant and diverse enzymes, responsible not only for protein degradation but also for regulating specific functional proteins through specificity to maintain the normal functioning of organisms.”

2. Lines 41-42: “Trypsin causes tissue damage due to protein hydrolysis, so trypsin is produced as an inactive precursor called trypsinogen.”

3. Lines 54-56: “A process mediated primarily by trypsin-catalyzed degradation of muscle tissue, which severely constrains shrimp shelf life during refrigerated transport.”

Materials and methods: The logical flow is acceptable; only English language editing is required.

Result: The logical flow is acceptable; only English language editing is required.

Discussion: Overall, the Discussion would be strengthened by clearer separation between data-supported conclusions and hypothesis-driven speculation, as well as by a more restrained interpretation aligned with the study’s experimental scope.

**Response to Reviewer #2**:

We sincerely thank the reviewer for the thorough evaluation and constructive suggestions. We have carefully addressed all comments and made substantial revisions to improve the manuscript quality.

**For Responding to English Language Quality:**

We acknowledge the reviewer’s concern about language clarity. In response:

**Native English editing**: We have engaged a native English-speaking colleague to comprehensively revise the manuscript, focusing on logical flow, sentence structure, and appropriate use of connectors.

**Specific revisions**: We have carefully revised all sentences highlighted by the reviewer in the Abstract and Introduction sections, improving: **1)** Logical connectors and sentence transitions, **2)** Wording precision and clarity, **3)** Sentence structure and coherence. **4)** In addition, we also proofread the full text of the manuscript.

**For Responding to Discussion Section:**

We sincerely appreciate this insightful comment. We fully agree that distinguishing between direct experimental evidence and theoretical implications is crucial for the scientific rigor of the manuscript. In the revised Discussion section, we have made the following adjustments:

**Clearer Separation**: We have restructured the text to explicitly distinguish conclusions directly derived from our results from broader hypotheses regarding their potential roles.

**Restrained Interpretation**: We have carefully refined the wording to avoid over-interpretation. Speculative statements have been tempered to ensure they strictly align with the experimental scope of this study.

**Supporting References**: We have added new relevant references to ground our hypothesis-driven speculations in established literature, ensuring that the discussion is both logical and well-supported.

**Linguistic Clarity**: Through the assistance of a native British colleague, we have optimized the logical connectors to clearly signal transitions between data analysis and scientific conjecture.
